# US Incidence of Late-Preterm Steroid Use and Associated Neonatal Respiratory Morbidity After Publication of the Antenatal Late Preterm Steroids Trial, 2015-2017

**DOI:** 10.1001/jamanetworkopen.2022.12702

**Published:** 2022-05-18

**Authors:** Mark A. Clapp, Alexander Melamed, Taylor S. Freret, Kaitlyn E. James, Cynthia Gyamfi-Bannerman, Anjali J. Kaimal

**Affiliations:** 1 Department of Obstetrics and Gynecology, Massachusetts General Hospital, Boston; 2 Harvard Medical School, Boston, Massachusetts; 3 New York–Presbyterian Hospital, Herbert Irving Comprehensive Cancer Center, Columbia University Vagelos College of Physicians and Surgeons, New York; 4 Department of Obstetrics and Gynecology, UC San Diego Health, San Diego, California

## Abstract

**Importance:**

The Antenatal Late Preterm Steroids (ALPS) trial demonstrated a 20% reduction in the risk of respiratory complications in neonates at risk for a late-preterm birth who were exposed to antenatal corticosteroids compared with those who were not.

**Objective:**

To assess whether new evidence of steroid administration for neonatal respiratory benefit in the late-preterm period is associated with changes in obstetric practice and the use of assisted ventilation for the neonate after delivery.

**Design, Setting, and Participants:**

This cross-sectional study of US births from February 1, 2015, to October 31, 2017, as ascertained from US natality data, included live-born, singleton neonates born between 34 and 36 completed weeks of gestation to people without pregestational diabetes. An interrupted time series analysis using Poisson regression models was conducted. Data were analyzed from July 11, 2022, to November 9, 2022.

**Exposures:**

Public dissemination of the ALPS trial results, which occurred during a 9-month period from February 1, 2016 (first published online), to October 31, 2016 (time of the last major professional society’s guideline update in the months after the trial’s publication).

**Main Outcomes and Measures:**

Steroid use, any assisted ventilation use, and assisted ventilation use for more than 6 hours immediately after the dissemination period.

**Results:**

A total of 707 862 births were included, divided among the 12-month predissemination period (n = 250 643), dissemination period (n = 195 736), and 12-month postdissemination period (n = 261 493). Most births were at 36 weeks of gestation (53.9% in the predissemination and postdissemination period; *P* = .10). Small but significant differences were found between the predissemination and postdissemination period cohorts: there were more individuals 35 years or older (19.5% vs 17.9%), fewer White individuals (67.8% vs 69.8%), and more publicly insured individuals (50.5% vs 50.1%) in the postdissemination period compared with the predissemination period, respectively (*P* < .001 for all). Compared with what rates were expected based on the predissemination trends, the adjusted rate of steroid use increased from 5.0% to 11.7% (adjusted incidence rate ratio [IRR], 2.34; 95% CI, 2.13-2.57), and assisted ventilation use decreased from 8.9% to 8.2% (adjusted IRR, 0.91; 95% CI, 0.85-0.98) after the dissemination period. No change was observed in assisted ventilation use for more than 6 hours (adjusted IRR, 0.98; 95% CI, 0.87-1.10).

**Conclusions and Relevance:**

These findings suggest that there was an immediate change in practice of administering antenatal steroids and a reduction in neonatal morbidity among late-preterm births associated with the dissemination of the ALPS trial, suggesting that this evidence may be translating into a reduction in immediate respiratory morbidity outside the context of a clinical trial.

## Key Points

**Question:**

Was the publication and dissemination of a randomized clinical trial showing neonatal benefit from the administration of corticosteroids to people at risk for preterm delivery between 34 and 36 completed weeks of gestation associated with a change in clinical practice and neonatal outcomes?

**Findings:**

In this cross-sectional study, the Antenatal Late Preterm Steroids trial was associated with an immediate increase in steroid use and a reduction in assisted ventilation use among late-preterm neonates in the US.

**Meaning:**

These findings suggest a benefit of antenatal late-preterm steroid use for neonatal respiratory morbidity outside the context of a clinical trial.

## Teaser

This cross-sectional study of neonates at risk for a late-preterm birth assesses whether new evidence on the benefit of steroid administration is associated with changes in clinical practice and neonatal outcomes.

## Introduction

Exposure to corticosteroids has been shown to decrease neonatal respiratory morbidity by stimulating surfactant production in the fetal lungs when administered antenatally before a preterm delivery.^1,2^ It also has been shown to reduce other neonatal complications of prematurity, especially in very preterm gestations.^3^ This practice has historically been limited to women at risk of delivering before 34 weeks of gestation based on the availability of existing data.^4,5^ In 2016, Gyamfi-Bannerman et al^6^ published the results of large-scale randomized clinical trial (the Antenatal Late Preterm Steroids [ALPS] trial) that evaluated the effects of steroid use in the late-preterm period (ie, 34-36 completed weeks of gestation). Infants born at these gestational ages are at higher risk of complications compared with full-term (≥37 weeks of gestation) neonates, especially regarding respiratory morbidity.^7,8^ The ALPS trial demonstrated a 20% reduction in the risk of respiratory complications in these late-preterm neonates who were randomized to antenatal steroids compared with those who were not.^6^

Shortly after the results of this clinical trial were presented and published, professional societies, including the Society for Maternal-Fetal Medicine and the American College of Obstetricians and Gynecologists, updated their clinical guidance to include consideration and/or recommendation for the administration of steroids to many people at risk of delivering in the late-preterm period.^9,10^ We sought to understand whether publication of this study and the related updates to clinical guidelines were associated with real-world practice changes and changes in respiratory outcomes for neonates in the late-preterm period. Leveraging a complete sample of late-preterm births in the US, we designed an interrupted time series study that evaluated the hypothesis that publication and dissemination of the ALPS trial findings would be associated with increased use of steroids in the late-preterm period and a corresponding reduction in respiratory morbidity in these neonates.

## Methods

For this cross-sectional study, we conducted an interrupted time series analysis using US natality data from February 1, 2015, to October 31, 2017.^11^ Data were obtained via publicly accessible files from the National Center for Health Statistics.^12^ The project was classified as non–human subjects research by the Mass General Brigham Human Subjects Research Committee and was therefore exempt from institutional review board approval and informed consent. This study followed the Strengthening the Reporting of Observational Studies in Epidemiology (STROBE) reporting guideline.^13^

Liveborn, singleton neonates with gestational ages between 34 and 36 completed weeks (ie, late-preterm births) born to women without pregestational diabetes were included in the study. This cohort was selected to approximate the population eligible for inclusion in the ALPS trial (ie, patients who had a singleton pregnancy, high probability of delivery in the late-preterm period, no history of pregestational diabetes, and no previous receipt of corticosteroids).^6^ Of note, the natality data do not provide length of gestation in days (only completed weeks), indications for delivery (eg, premature rupture of membranes), or data on length of stay or any potential antepartum hospital course.

Findings of the ALPS trial were first presented and published online in February 2016.^6,14^ The trial results were then published in print in April 2016.^6^ Two of the major obstetric professional societies, the Society for Maternal-Fetal Medicine and the American College of Obstetricians and Gynecologists, revised their guidance to obstetric practitioners to incorporate the ALPS trial evidence within the following months (August and October 2016, respectively).^9,10^ Thus, we considered these 9 months (February 1 to October 31, 2016) to be the ALPS trial dissemination period in which practitioners learned of the recent evidence and updates to clinical practice recommendations regarding the benefits of administering corticosteroids to certain patients at risk of a late-preterm delivery.

To conduct the interrupted time series analysis, we a priori selected a 12-month observational period before (February 1, 2015, to January 30, 2016) and after (November 30, 2016, to October 31, 2017) the dissemination period to quantify whether the trial was associated with changes in obstetric practice and neonatal outcomes. This follow-up period (12 months) was selected to maximize the likelihood that the following assumptions of an interrupted time series were met: (1) there was linearity in the preintervention trend, (2) the characteristics of the patients did not change throughout the study period, and (3) no other intervention simultaneously affected the measured outcomes within the study cohort.^11^ The following patient characteristics were compared between the predissemination and postdissemination groups using χ^2^ tests: gestational age (categorized as 34, 35, or 36 completed weeks), maternal age (categorized as <18, 18-24, 25-29, 30-34, 35-39, or ≥40 years), mother’s designated race using the standard US natality recoding definitions (American Indian or Alaska Native, Asian, Black, Native Hawaiian or Other Pacific Islander, White, or >1 race), mother’s designated ethnicity using the standard US natality recoding definition (Hispanic or non-Hispanic), primary payer for delivery admission (categorized as Medicaid, private, self-pay, or other), and delivering practitioner (categorized as physician, midwife, or other). Race and ethnicity were included for comparison because prior reports^15,16^ have noted differential rates of steroid administration and neonatal outcomes among these groups.

The primary outcome of interest was the use of immediate assisted ventilation after delivery. As a secondary outcome, we examined assisted ventilation use for more than 6 hours, which occurred in a subgroup of neonates with the primary outcome. These outcomes were chosen for the following reasons: (1) they are discretely collected on the US Standard Certificate of Birth (2003 revised version); (2) there is preestablished mechanistic probability for antenatally administered corticosteroids to affect neonatal respiratory morbidity; and (3) these outcomes were most relevant to the primary outcome from the ALPS trial, which was a composite of continuous partial airway pressure of 2 hours or more, fraction of inspired oxygen of 0.30 or more for 4 hours or more, mechanical ventilation, extracorporeal membrane oxygenation, or stillbirth or neonatal death at 72 hours or less.^6^ The definitions for each outcome used in this study, as they appear in the Centers for Disease Control and Prevention’s instructions for completing the certificate of birth, are listed in the eMethods in the Supplement.^17^ We examined changes in corticosteroid administration to demonstrate whether publication and dissemination of ALPS trial findings were associated with changes in obstetric practice.

### Statistical Analysis

Because we hypothesized that the adoption of late-preterm corticosteroid administration continued after the dissemination period, we selected an impact model that allowed for an immediate level change after the dissemination period (the parameter of interest) as well as a change in trend. We fit Poisson regression models that estimated the relative risk of each outcome for a delivery that occurred immediately after the dissemination period relative to a counterfactual model based on extrapolating the outcome trends observed in the 12 months preceding the dissemination period. A full description of the interrupted time series model is included in the eMethods in the Supplement. We fit both crude and adjusted models in a complete case analysis, which included gestational age, maternal age, race, ethnicity, primary payer for delivery admission, and primary delivering practitioner. Covariates were selected a priori based on factors that have been previously demonstrated or that were hypothesized to be related to antenatal steroid administration or neonatal respiratory outcomes.^15,16,18,19^ In all models, SEs were estimated by bootstrapping with replacement with 50 replications.

The robustness of the primary findings was evaluated using placebo tests in time.^20^ Specifically, we fit the same time series models (with a 12-month predissemination period, 9-month dissemination period, and 12-month postdissemination period) in periods before and after (but not overlapping with) the ALPS trial dissemination period, in which no change in practice or outcomes was expected.^21^ A total of 24 placebo tests (12 before and 12 after the dissemination period) were performed and used to generate an empirical distribution of the relative risk estimates under the null hypothesis of no association. This distribution was used to evaluate whether an association observed in the true interrupted time series was larger than one measured in a period chosen at random. This placebo test follows the logic of a permutation test, such as the Fisher exact test.^22^ A full description and visualization of the placebo testing is included in the eMethods in the Supplement.

To further evaluate the sensitivity of our findings to model specifications, we evaluated the use of longer and shorter predissemination and postdissemination periods in the time series models. Follow-up windows were shortened (9 months) and lengthened (18 and 24 months) with the otherwise same specifications as the primary model. A full description and visualization of this robustness check is included in the eMethods in the Supplement.

Statistical analysis was conducted from July 11, 2022, to November 9, 2022. Analyses were performed using Stata MP software, version 16.0 (StataCorp LLC). A 2-sided *P* < .05 was considered statistically significant.

## Results

A total of 10 694 111 births occurred between February 1, 2015, and October 31, 2017, of which 862 006 (8.1%) were between 34 and 36 completed weeks of gestation. After multiple births (n = 134 445) and women with pregestational diabetes (n = 15 867) were excluded for similarity to the original trial, a total of 707 862 births were included, divided among the predissemination period (n = 250 643), dissemination period (n = 195 736), and postdissemination period (n = 261 493). eFigure 1 in the Supplement shows the CONSORT diagram for the population included.

Table 1 compares the characteristics of the study population in the predissemination and postdissemination periods. No differences were found in the distribution of gestational ages and maternal ethnicity in the late-preterm period between the cohorts. Most neonates in both cohorts were born at 36 completed weeks of gestation (53.9% in both). Small but significant differences were found between the 2 cohorts: more individuals 35 years or older (19.5% vs 17.9%), fewer White individuals (67.8% vs 69.8%), and more publicly insured individuals (50.5% vs 50.1%) in the postdissemination period compared with the predissemination period, respectively (*P* < .001 for all).

**Table 1.** Comparison of Patient Characteristics Before and After the Antenatal Late Preterm Steroids Trial Dissemination

Characteristic	No. (%) of patients		*P* value
	Predissemination (n = 250 643)	Postdissemination (n = 261 493)	
Gestational age, completed wk			
34	79 796 (17.9)	47 162 (18.0)	.10
35	126 174 (28.3)	73 413 (28.1)	
36	240 409 (53.9)	140 918 (53.9)	
Maternal age, y			
≤18	5515 (2.2)	4914 (1.9)	<.001
19-24	69 383 (27.7)	66 655 (25.5)	
25-29	67 909 (27.1)	71 338 (27.3)	
30-34	63 040 (25.2)	67 599 (25.9)	
35-39	34 891 (13.9)	39 841 (15.2)	
≥40	9905 (4.0)	11 146 (4.3)	
Race			
American Indian or Alaska Native	3140 (1.3)	3242 (1.2)	<.001
Asian	13 974 (5.6)	16 417 (6.3)	
Black	51 306 (20.5)	56 708 (21.7)	
Native Hawaiian or Other Pacific Islander	1091 (0.4)	1046 (0.4)	
White	174 898 (69.8)	177 314 (67.8)	
>1 Race	6234 (2.5)	6766 (2.6)	
Ethnicity			
Hispanic	61 860 (24.7)	65 064 (24.9)	.08
Missing	1841 (0.7)	2022 (0.8)	
Payer			
Medicaid	125 644 (50.1)	132 110 (50.5)	<.001
Private	103 324 (41.2)	107 942 (41.3)	
Self-pay	9231 (3.7)	9879 (3.8)	
Other	10 547 (4.2)	9978 (3.8)	
Missing	1897 (0.8)	1584 (0.6)	
Delivery practitioner			
Physician	232 775 (92.9)	242 348 (92.7)	<.001
Certified nurse midwife	15 538 (6.2)	16 803 (6.4)	
Other	2238 (0.9)	2216 (0.8)	
Missing	92 (<1)	126 (<1)	

**Figure 1.**
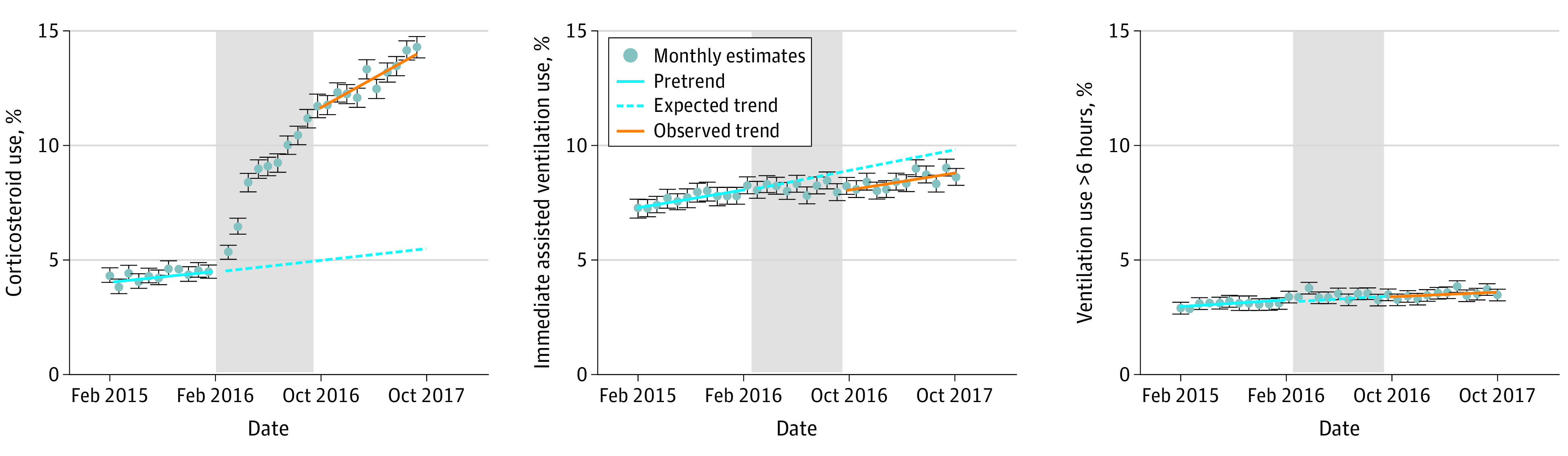
Adjusted Trends for Steroid and Assisted Ventilation Use Before and After the Antenatal Late Preterm Steroids Trial Dissemination Period The adjusted models included the following covariates: completed weeks of gestation, maternal age, maternal race, maternal ethnicity, primary payer for birth encounter, and delivering practitioners. The gray shaded area represents the dissemination period (February to October 2016).

Figure 1 shows the monthly adjusted rates of steroid use, immediate assisted ventilation use, and ventilation use for more than 6 hours during the predissemination, dissemination, and postdissemination periods. The figure illustrates the association between the ALPS trial dissemination and the trajectory of monthly steroid and assisted ventilation rates. In the month after the ALPS dissemination period, the adjusted rate of steroid administration was significantly higher than expected based on the trends preceding this period (11.7% observed vs 5.0% expected; unadjusted incidence rate ratio [IRR], 2.35; 95% CI, 2.18-2.55; adjusted IRR, 2.34, 95% CI, 2.13-2.57). Similarly, ALPS dissemination was associated with a decrease in the adjusted rate of assisted ventilation among late-preterm neonates (8.2% observed vs 8.9% expected; unadjusted IRR, 0.93; 95% CI, 0.86-1.00; adjusted IRR, 0.91; 95% CI, 0.85-0.98). No association was observed between dissemination of ALPS trial findings and the rate of assisted ventilation use for more than 6 hours (3.4% observed vs 3.4% expected; unadjusted IRR, 0.99; 95% CI, 0.88-1.12; adjusted IRR, 0.98; 95% CI, 0.87-1.10).

**Figure 2.**
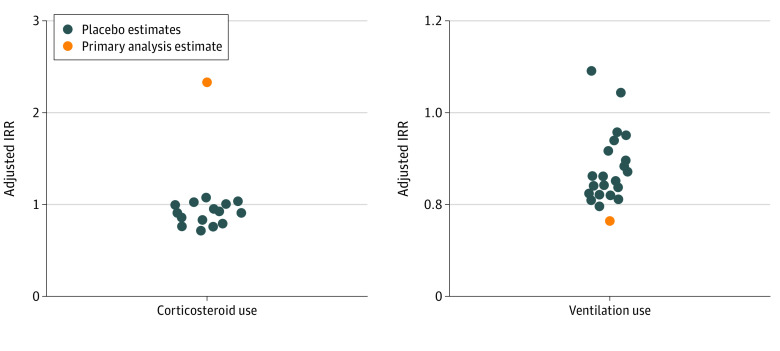
Adjusted Incidence Rate Ratio (IRR) Estimates From the Placebo Tests Compared With the Primary Analysis The adjusted models included the following covariates: completed weeks of gestation, maternal age, maternal race, maternal ethnicity, primary payer for birth encounter, and delivering practitioner.

Results from the placebo tests, which assumed 24 false pretrial, dissemination, and posttrial periods, are shown in Figure 2. For steroid administration and use of assisted ventilation, the magnitude of the association measured in the true interrupted time series model was larger than that seen in any of the placebo models, suggesting that the observed associations in the true model were less likely due to chance alone or model misspecification. Full results from the adjusted models are included in eTable 1 in the Supplement.

Table 2 summarizes the results from the robustness test of the a priori–determined predissemination and postdissemination period lengths (12 months). Adjusted IRRs are reported for 3 alternative interrupted time series models in which the observation period was shortened (9-month follow-up period) and lengthened (18-month and 24-month follow-up periods). eFigure 2 in the Supplement shows the monthly adjusted rates of steroid use, immediate assisted ventilation use, and ventilation use for more than 6 hours with the varied follow-up periods. Both crude and adjusted IRRs for change in steroid administration and assisted ventilation rates are given in eTables 2, 3, and 4 in the Supplement. The association of the ALPS trial dissemination with steroid use remained similar regardless of the follow-up window. The association between trial dissemination and immediate assisted ventilation use was not significant when the 9-month observation period was used, although the point estimate was comparable; however, the CI around the observed point estimate (adjusted RR, 0.91) tightened as the sample size increased when the observation period was extended to 12, 18, and 24 months. Similar findings were seen for assisted ventilation of more than 6 hours after birth, although these findings were not statistically significant (24-month adjusted IRR, 0.94; 95% CI, 0.88-1.01).

**Table 2.** Adjusted IRRs for Steroid and Assisted Ventilation Use With Varying Predissemination and Postdissemination Period Lengths^a^

Measure	Adjusted IRR (95% CI)			
	9 Mo	12 Mo (primary analysis)	18 Mo	24 Mo
Steroid use	2.32 (2.07-2.60)	2.34 (2.13-2.57)	2.45 (2.32-2.59)	2.48 (2.38-2.60)
Immediate assisted ventilation use	0.94 (0.87-1.03)	0.91 (0.85-0.98)	0.91 (0.87-0.96)	0.92 (0.88-0.96)
Ventilation use >6 h	1.03 (0.88-1.20)	0.98 (0.87-1.10)	0.95 (0.87-1.04)	0.94 (0.88-1.01)

Abbreviation: IRR, incidence rate ratio.

^a^ The predissemination and postdissemination periods were varied (9, 18, and 24 months) compared with the a priori–selected primary analysis follow-up period (12 months). The adjusted models included the following covariates: completed weeks of gestation, maternal age, maternal race, maternal ethnicity, primary payer for birth encounter, and delivering practitioner.

## Discussion

Using a robust, quasi-experimental interrupted time series design and a complete sample of late-preterm births in the US, this study demonstrates that dissemination of the ALPS trial was associated with changes in obstetric practice and neonatal outcomes. Steroid administration among infants born in the late-preterm period more than doubled in the 9 months after the publication of the study,^6^ and assisted ventilation among the infants decreased by 9%. These findings were robust to various sensitivity analyses, and the adjustment for multiple potential confounders only reduced the uncertainty of the estimates, strengthening our confidence in the validity of the results.

In the multicenter randomized clinical trial of 2831 women without pregestational diabetes and with expected delivery between 24 hours and 7 days at time of recruitment, the ALPS study^6^ demonstrated a reduction of the primary respiratory composite outcome among neonates exposed to corticosteroids. This study provided convincing evidence that corticosteroid administration in late-preterm infants reduces respiratory morbidity, prompting professional societies to change their clinical practice recommendations. The primary ALPS trial outcome was reflective of significant respiratory morbidity, although most of the composite was driven by the use of continuous positive air pressure or high-flow nasal cannula use for 2 hours or longer. In our study, we were able to observe assisted ventilation use for more than 6 hours, which is also likely reflective of significant respiratory morbidity. However, markers of respiratory morbidity were not directly measured at this time point in the ALPS trial. As a secondary outcome in the ALPS trial, the need for resuscitation at birth was reported to be significantly lower in the steroid-exposed group. This outcome is most similar to our study’s primary outcome (immediate assisted ventilation use), in which we also detected a significant reduction after the trial’s dissemination.

Although randomized clinical trials are often considered the criterion standard for determining causality, their translation into clinical practice can be limited because of issues with generalizability.^23,24,25^ The use of quasi-experimental methods, such as an interrupted time series analysis, can allow us to examine associations between the dissemination of new evidence and practice changes. In our study, we report an increase in steroid use and a decrease in immediate neonatal ventilation use after the release of new evidence without being able to discern the exact timing of steroid administration, the clinical circumstances in which they were administered, or the type and exact duration of assisted ventilation use; these findings enhance the external validity and generalizability of the ALPS trial’s findings. Furthermore, this practice change occurred more rapidly than has previously been observed for other evidenced-based adoptions of clinical and translational research.^26^ We hypothesize that this adoption may be related to the fact that this new evidence was a direct extension of an identical intervention for women with expected preterm deliveries at earlier than 34 weeks of gestation, increasing practitioner familiarity and comfort in adopting the recommendation.

### Strengths and Limitations

The strengths of this study include its use of a complete sample of late-preterm births in the US, its quasi-experimental design, and multiple robustness checks. The US birth certificate data are the only single, available national data source in which gestational age, linked maternal information (ie, steroid administration), and neonatal outcomes can be used to examine this question at a population level. The variables that are uniformly collected on the 2003 revised version of the US Standard Certificate of Birth (“steroids [glucocorticoids] for fetal lung maturation received by the mother prior to delivery,” “assisted ventilation required immediately following delivery,” and “assisted ventilation required for more than six hours”) are not identical, although they serve as close proxies to the exposure or primary outcomes evaluated in the ALPS trial.

Our study should be interpreted with the following limitations. Prior reports^27,28,29^ have raised concerns about the validity of certain birth certificate data elements and noted the low sensitivity and state-level variation of birth certificates for steroid administration and ventilation use. Low sensitivity would result in an underestimation of the true rates. However, as long as dissemination of the ALPS trial did not also coincide with simultaneous changes in the birth certificate reporting process, the relative changes (as measured by the IRRs) are meaningful and interpretable. The largest threat to the validity of our findings is the possibility that other factors, such as a different change in clinical practice, coincided with the ALPS trial and that this concurrent change may have affected real or reported rates of assisted ventilation. We believe that this possibility is unlikely for several reasons. First, we are unaware of any simultaneous recommendations or policy changes that could have plausibly affected neonatal respiratory outcomes during the study period. Second, we found that the decrease in the risk of assisted ventilation associated with the ALPS dissemination period was large compared with changes in this outcome observed in periods selected at random (ie, placebo testing); this finding suggests that changes in the rate of assisted ventilation that may result from unknown factors, or chance alone, are usually smaller in magnitude than the association observed in this interrupted time series.

More detailed information on the exposure and outcomes (eg, exact timing, indications, and duration) would ideally be helpful in better isolating the effectiveness of this intervention, although such types of information are rarely available in population-based analyses. Of importance, we were unable to assess other short-term (eg, neonatal hypoglycemia) or long-term (eg, neurodevelopmental) outcomes of corticosteroid administration in the late-preterm period, which have been previously reported.^6,10,30,31,32^ Any potential benefit should be considered in tandem of potential reported risks.

## Conclusions

In conclusion, this cross-sectional study found that the publication and dissemination of the ALPS trial data were associated with an immediate increase in the use of steroids among late-preterm births in the US. Correspondingly, there was a reduction in immediate assisted ventilation use, a finding that is consistent with and provides evidence of generalizability of the primary clinical trial’s results.^6^ Future research on the effects on neonatal intensive care resource use, the rates of neonatal hypoglycemia, and the appropriateness of steroid administration use in the post–ALPS trial period is needed for a holistic understanding of the effects of antenatally administered steroids in the late-preterm period.

## Notes

Supplement. **eMethods.** Supplement Methods **eFigure 1.** CONSORT Diagram **eFigure 2**. Adjusted Trends for Steroid and Assisted Ventilation Use Before and After the Antenatal Late Preterm Steroid Trial Dissemination Period With Varying Pre- and Post-Dissemination Follow-up Periods **eTable 1.** Adjusted Incidence Rate Ratio Estimates for the Placebo Tests in Comparison to the Effect Estimated in the Main Analysis **eTable 2.** Unadjusted and Adjusted Incidence Rate Ratios for Steroid Use With Varying Pre- and Post- Dissemination Follow-up Periods **eTable 3.** Unadjusted and Adjusted Incidence Rate Ratios for Immediate Assisted Ventilation Use With Varying Pre- and Post-Dissemination Follow-up Periods **eTable 4.** Unadjusted and Adjusted Incidence Rate Ratios for Assisted Ventilation Use >6 Hours With Varying Pre- and Post-Dissemination Follow-up Periods
