# Supplementary material for: US Incidence of Late-Preterm Steroid Use and Associated Neonatal Respiratory Morbidity After Publication of the Antenatal Late Preterm Steroids Trial, 2015-2017
Source: JAMA Netw Open. 2022 May 18;5(5):e2212702. doi: 10.1001/jamanetworkopen.2022.12702 (PMC9118048; doi:10.1001/jamanetworkopen.2022.12702)
Supplement: Supplement. — eMethods. Supplement Methods eFigure 1. CONSORT Diagram eFigure 2. Adjusted Trends for Steroid and Assisted Ventilation Use Before and After the Antenatal Late Preterm Steroid Trial Dissemination Period With Varying Pre- and Post-Dissemination Follow-up Periods eTable 1. Adjusted Incidence Rate Ratio Estimates for the Placebo Tests in Comparison to the Effect Estimated in the Main Analysis eTable 2. Unadjusted and Adjusted Incidence Rate Ratios for Steroid Use With Varying Pre- and Post- Dissemination Follow-up Periods eTable 3. Unadjusted and Adjusted Incidence Rate Ratios for Immediate Assisted Ventilation Use With Varying Pre- and Post-Dissemination Follow-up Periods eTable 4. Unadjusted and Adjusted Incidence Rate Ratios for Assisted Ventilation Use >6 Hours With Varying Pre- and Post-Dissemination Follow-up Periods [file jamanetwopen-e2212702-s001.pdf]

Supplemental Online Content

Clapp MA, Melamed A, Freret TS, James KE, Gyamfi-Bannerman C, Kaimal AJ. US incidence of late-preterm steroid use and associated neonatal respiratory morbidity after publication of the Antenatal Late Preterm Steroids Trial, 2015-2017. *JAMA Netw Open*. 2022;5(5):e2212702. doi:10.1001/jamanetworkopen.2022.12702

eMethods. Supplement Methods

eFigure 1. CONSORT Diagram

eFigure 2. Adjusted Trends for Steroid and Assisted Ventilation Use Before and After the Antenatal Late Preterm Steroid Trial Dissemination Period With Varying Pre- and Post-Dissemination Follow-up Periods

eTable 1. Adjusted Incidence Rate Ratio Estimates for the Placebo Tests in Comparison to the Effect Estimated in the Main Analysis

eTable 2. Unadjusted and Adjusted Incidence Rate Ratios for Steroid Use With Varying Pre- and Post-Dissemination Follow-up Periods

eTable 3. Unadjusted and Adjusted Incidence Rate Ratios for Immediate Assisted Ventilation Use With Varying Pre- and Post-Dissemination Follow-up Periods

eTable 4. Unadjusted and Adjusted Incidence Rate Ratios for Assisted Ventilation Use >6 Hours With Varying Pre- and Post-Dissemination Follow-up Periods

This supplemental material has been provided by the authors to give readers additional information about their work.

<u>eMethods. Supplemental Methods</u>

Data Set

The primary data source was deidentified birth certificate data, which was publicly available and downloaded directly from the National Center for Health Statistics.[1] It comprises comprehensive birth certificate information from all deliveries in the United States.

The following births were excluded for similarity to the original Antenatal Late Preterm Steroid (ALPS) trial: 1) those with gestational ages <34 and >36 weeks of gestation or unknown gestational age; 2) births from multiple gestations; and 3) births to patients with pre-gestational diabetes.[2] Some variables required for the analysis were only available on the 2003 revised version of the US Standard Certificate of Birth.[3] As of January 1, 2016, all states and territories had adopted this version.[4] Reporting flags for individual data elements were used in accordance with yearly Data Use Guidelines to ensure the reliability the included data.

Exposure and Outcome Definitions

The “Guide to Completing the Facility Worksheets for the Certificate of Live Birth and Report of Fetal Death” was used to identify the variables of interest for this analysis.[5]

Under “45. Characteristics of Labor and Delivery,” the item “Steroids (glucocorticoids) for fetal lung maturation received by mother prior to delivery” was used to observe steroid administration (see item as it appears on the US Standard Certificate of Birth in adjacent image). The definition for this item in the guidebook is listed as “*Steroids received by the mother prior to delivery to accelerate fetal lung maturation. Typically administered in anticipation of preterm (less than 37 completed weeks of gestation) delivery. Steroids include: betamethasone, dexamethasone, or hydrocortisone specifically given to accelerate fetal lung maturation.*” It instructs personnel completing the Certificate of Birth to “*Check this item when 1) steroid medication was given to the mother 2) prior to delivery [including prior to admission for delivery] 3) for fetal lung maturation.*”[5]

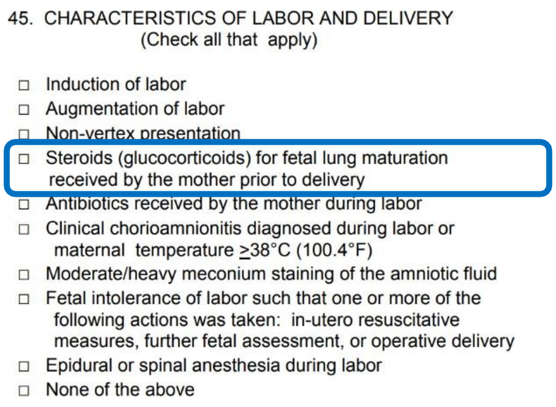
45. CHARACTERISTICS OF LABOR AND DELIVERY
(Check all that apply)

- ☐ Induction of labor
- ☐ Augmentation of labor
- ☐ Non-vertex presentation
- ☐ Steroids (glucocorticoids) for fetal lung maturation received by the mother prior to delivery
- ☐ Antibiotics received by the mother during labor
- ☐ Clinical chorioamnionitis diagnosed during labor or maternal temperature ≥38°C (100.4°F)
- ☐ Moderate/heavy meconium staining of the amniotic fluid
- ☐ Fetal intolerance of labor such that one or more of the following actions was taken: in-utero resuscitative measures, further fetal assessment, or operative delivery
- ☐ Epidural or spinal anesthesia during labor
- ☐ None of the above

The neonatal respiratory outcomes were immediate assisted ventilation use after delivery and assisted ventilation use >6 hours (see items as they appear on the on the US Standard Certificate of Birth in adjacent image). The definition for immediate assisted ventilation in the guidebook is listed as “*Infant given manual breaths for any duration with bag and mask or bag and endotracheal tube within the first several minutes from birth. Excludes free-flow (blow-by) oxygen only, laryngoscopy for aspiration of meconium, nasal cannula, and bulb suction.*”[5] The definition for assisted ventilation for more than 6 hours in the guidebook is listed as “*Infant given mechanical ventilation (breathing assistance) by any method for more than 6 hours. Includes conventional, high frequency, or continuous positive pressure (CPAP). Excludes free-flow oxygen only, laryngoscopy for aspiration of meconium, and nasal cannula.*”[5]

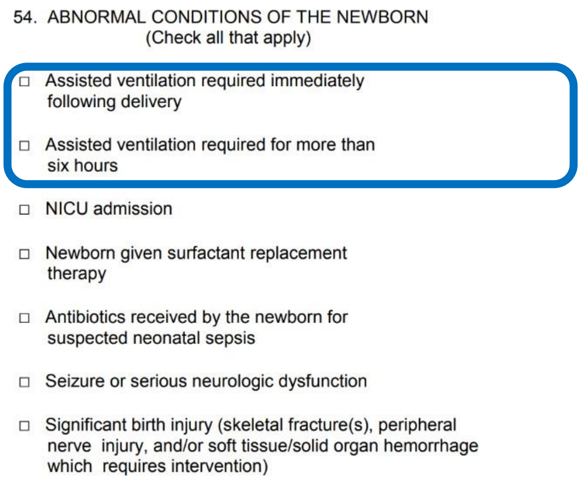
54. ABNORMAL CONDITIONS OF THE NEWBORN
(Check all that apply)

- ☐ Assisted ventilation required immediately following delivery
- ☐ Assisted ventilation required for more than six hours
- ☐ NICU admission
- ☐ Newborn given surfactant replacement therapy
- ☐ Antibiotics received by the newborn for suspected neonatal sepsis
- ☐ Seizure or serious neurologic dysfunction
- ☐ Significant birth injury (skeletal fracture(s), peripheral nerve injury, and/or soft tissue/solid organ hemorrhage which requires intervention)

© 2022 Clapp MA et al. *JAMA Network Open.*

Primary Interrupted Time Series Analysis

An interrupted time series analysis was conducted to determine the immediate effect of the ALPS trial on steroid administration and assisted ventilation.[2]

Poisson models were constructed using the following equation to generate relative changes in rates for the variables of interest:

$$Y_{!} = \beta_{"} + \beta_{\#} T + \beta_{\$} X_{!} + \beta_{\%} T X_{!} + B_{\&} X_{'} + \varepsilon$$

where
- $Y_{!}$: outcome at time *t*
- T: time in months
- $X_{!}$: indicator variable for the pre- or post-dissemination period
- $X_{'}$: patient-specific factors, *adjusted model only*
- ε: error term, estimated from bootstrapping *(n=50 iterations)*

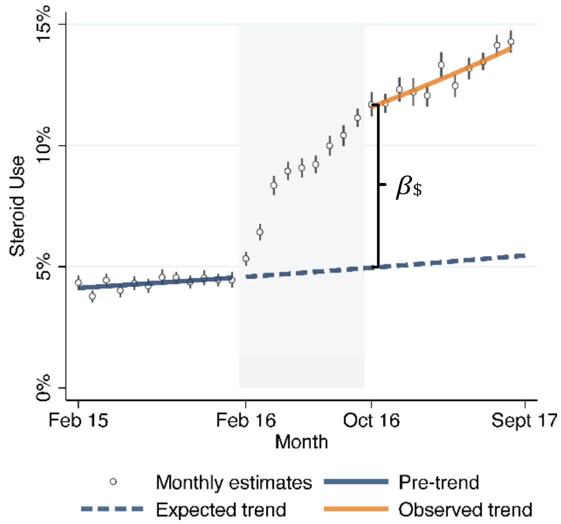

$\beta_{\$}$ represents the immediate effect of the intervention on the outcome. Incidence rate ratios reported are for the $\beta_{\$}$ coefficient.

Patient-specific factors were added in the adjusted model and included:
- Gestational age (categorized as 34, 35, 36 completed weeks)
- Maternal age (categorized as <18, 18-24, 25-29, 29-34, 35-29, ≥40 years)
- Maternal race (categorized as White, Black, Asian, American Indian/Alaskan Native, Native Hawaiian or Other Pacific Islander, other or more than one race)
- Maternal ethnicity (categorized as Hispanic or non-Hispanic)
- Primary payer for delivery encounter (Medicaid, private insurer, self-pay, other)
- Delivering provider (physician, certified nurse midwife, other)

The time periods were designated as follows:
- Pre-period: 12 months, February 2015-January 2016
- Trial dissemination period (or washout period): 9 months (February 2016-October 2016) ranging from the study's first publication online at nejm.org to the publication of the American College of Obstetricians and Gynecologists' (ACOG) Practice Bulletin 171 (the last updated to major societal practice guidelines in the initial months following the trial publication)[2,6–8]
- Post-period: 12 months, November 2016-October 2017

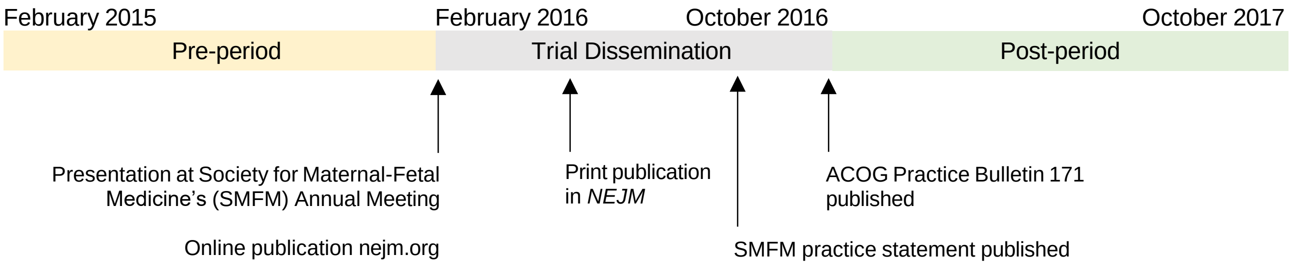

© 2022 Clapp MA et al. *JAMA Network Open.*

Robustness Checks

Two methods were used to evaluate the robustness of the primary findings:

1. Placebo Testing

First, we conducted “placebo” tests, in which artificial dissemination periods and subsequent pre-period and post-policy periods were constructed prior to and following the true dissemination period. We advanced the model forward or backward by one month for a year, avoiding any overlap with the true dissemination period, which resulted in in a total of 24 placebo tests (shown below). There were no expected changes in steroid use or neonatal respiratory morbidity in these placebo test periods. Adjusted Poisson models with the same model specifications as the main analysis were used to generate an empirical distribution of incidence rate ratios for steroid use and immediate assisted ventilation use under the null hypothesis of no association. Placebo testing was not done for assisted ventilation use >6 hours, as this outcome was not significant in the main analysis.

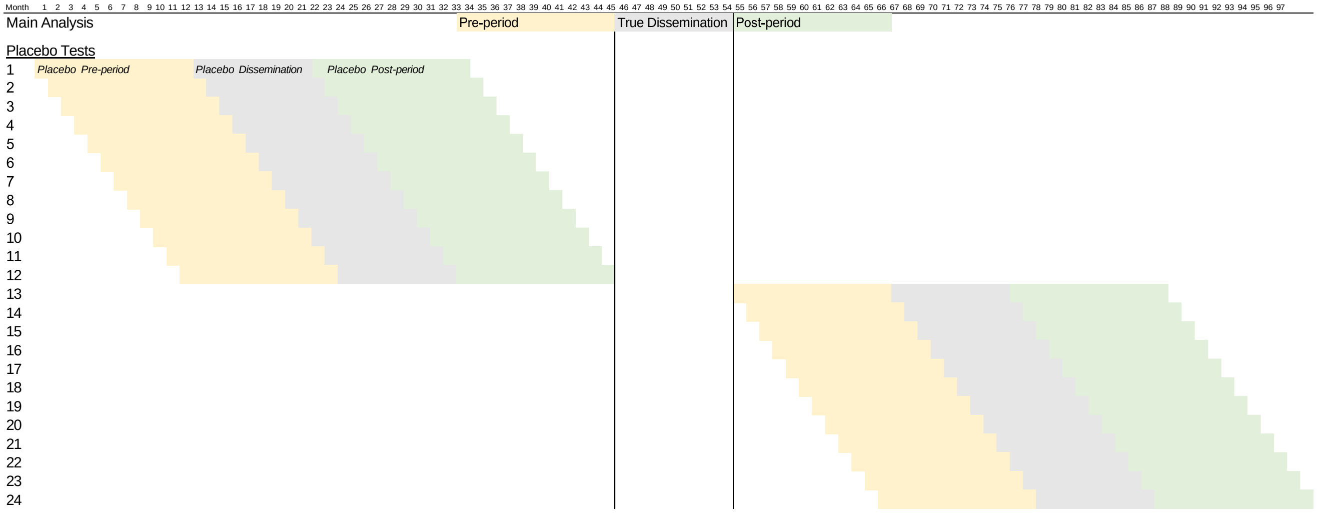

Point estimates were compared among the placebo tests and the main analysis estimate to evaluate whether the observed association in the true interrupted time series analysis was significantly different than one measured at a time chosen at random (eTable 1 and Figure 2). Fisher’s Exact test was used to determine the significance of the observed main effect in relation to the placebo tests.

2. Varying Lengths of Follow-up Period

The length of the pre- and post-periods (12 months) was selected a priori. It was chosen to be longer than the dissemination period, but not so long that outlying points affected by other clinical practice changes or temporal trends would modify the true association.

As a comparison to the main analysis, we performed the same unadjusted and adjusted Poisson models but varied the follow-up lengths (9 months, 18 months, 24 months).

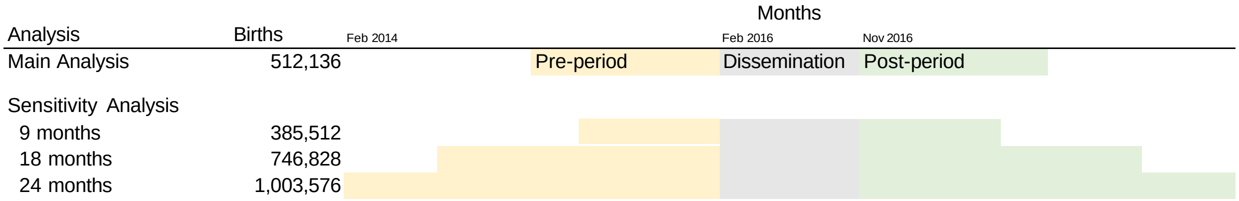

**Birth totals exclude the dissemination period.*

Results from these varying follow-up periods are shown in Table 3, eFigure 3, and eTables 2-4.

© 2022 Clapp MA et al. *JAMA Network Open.*

eReferences

1. NVSS - Birth Data. Published September 24, 2021. Accessed October 29, 2021. https://www.cdc.gov/nchs/nvss/births.htm

2. Gyamfi-Bannerman C, Thom EA, Blackwell SC, et al. Antenatal Betamethasone for Women at Risk for Late Preterm Delivery. *New England Journal of Medicine*. 2016;374(14):1311-1320. doi:10.1056/NEJMoa1516783

3. Centers for Disease Control and Prevention (CDC). Revisions of the U.S. Standard Certificates and Reports. National Center for Health Statistics. Published August 30, 2017. Accessed April 11, 2019. https://www.cdc.gov/nchs/nvss/revisions-of-the-us-standard-certificates-and-reports.htm

4. About NCHS - NCHS Fact Sheets - National Vital Statistics System Improvements. Published June 17, 2021. Accessed November 4, 2021. https://www.cdc.gov/nchs/about/factsheets/factsheet_nvss_improvements.htm

5. Centers for Disease Control and Prevention (CDC). Guide to Completing the Facility Worksheets for the Certificate of Live Birth and Report of Fetal Death. National Center for Health Statistics. Published September 4, 2019. Accessed November 23, 2021. https://www.cdc.gov/nchs/nvss/facility-worksheets-guide.htm

6. Practice Bulletin No. 171: Management of Preterm Labor. *Obstet Gynecol*. 2016;128(4):e155-164. doi:10.1097/AOG.0000000000001711

7. Gyamfi-Bannerman C. 1: Antenatal Late Preterm Steroids (ALPS): a randomized trial to reduce neonatal respiratory morbidity. *American Journal of Obstetrics & Gynecology*. 2016;214(1):S2. doi:10.1016/j.ajog.2015.10.022

8. Society for Maternal-Fetal Medicine (SMFM) Publications Committee. Implementation of the use of antenatal corticosteroids in the late preterm birth period in women at risk for preterm delivery. *Am J Obstet Gynecol*. 2016;215(2):B13-15. doi:10.1016/j.ajog.2016.03.013

© 2022 Clapp MA et al. *JAMA Network Open.*

eFigure 1: CONSORT diagram

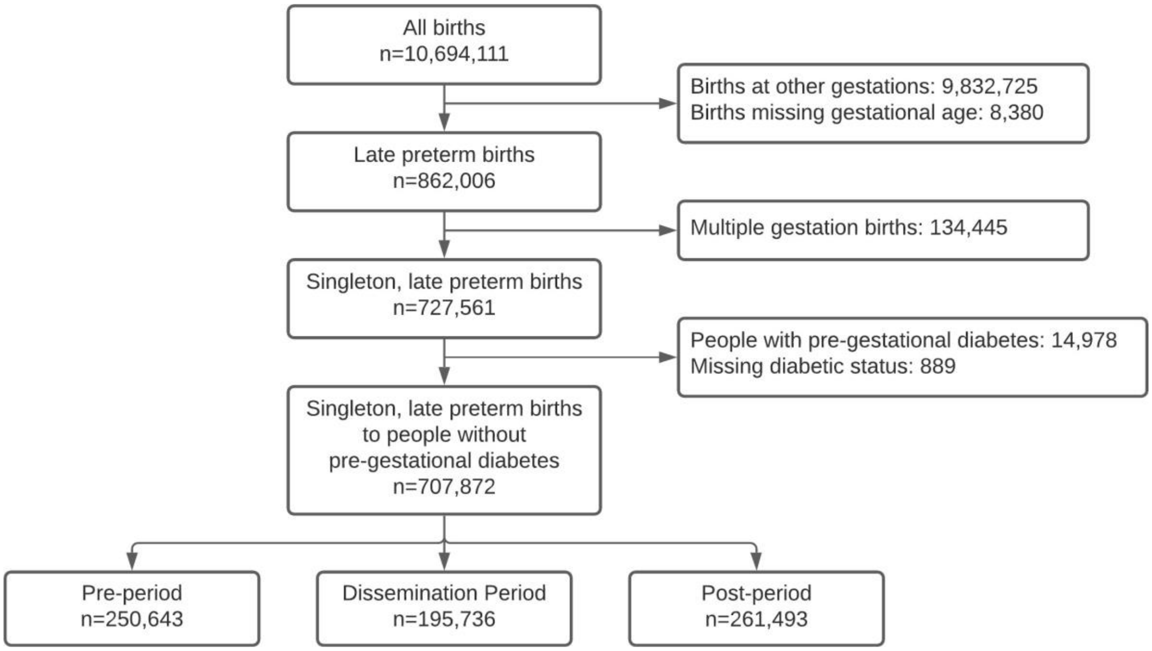

© 2022 Clapp MA et al. *JAMA Network Open.*

eFigure 2: Adjusted trends for steroid and assisted ventilation use before and after the Antenatal Late Preterm Steroid trial dissemination period with varying pre- and post-dissemination follow-up periods

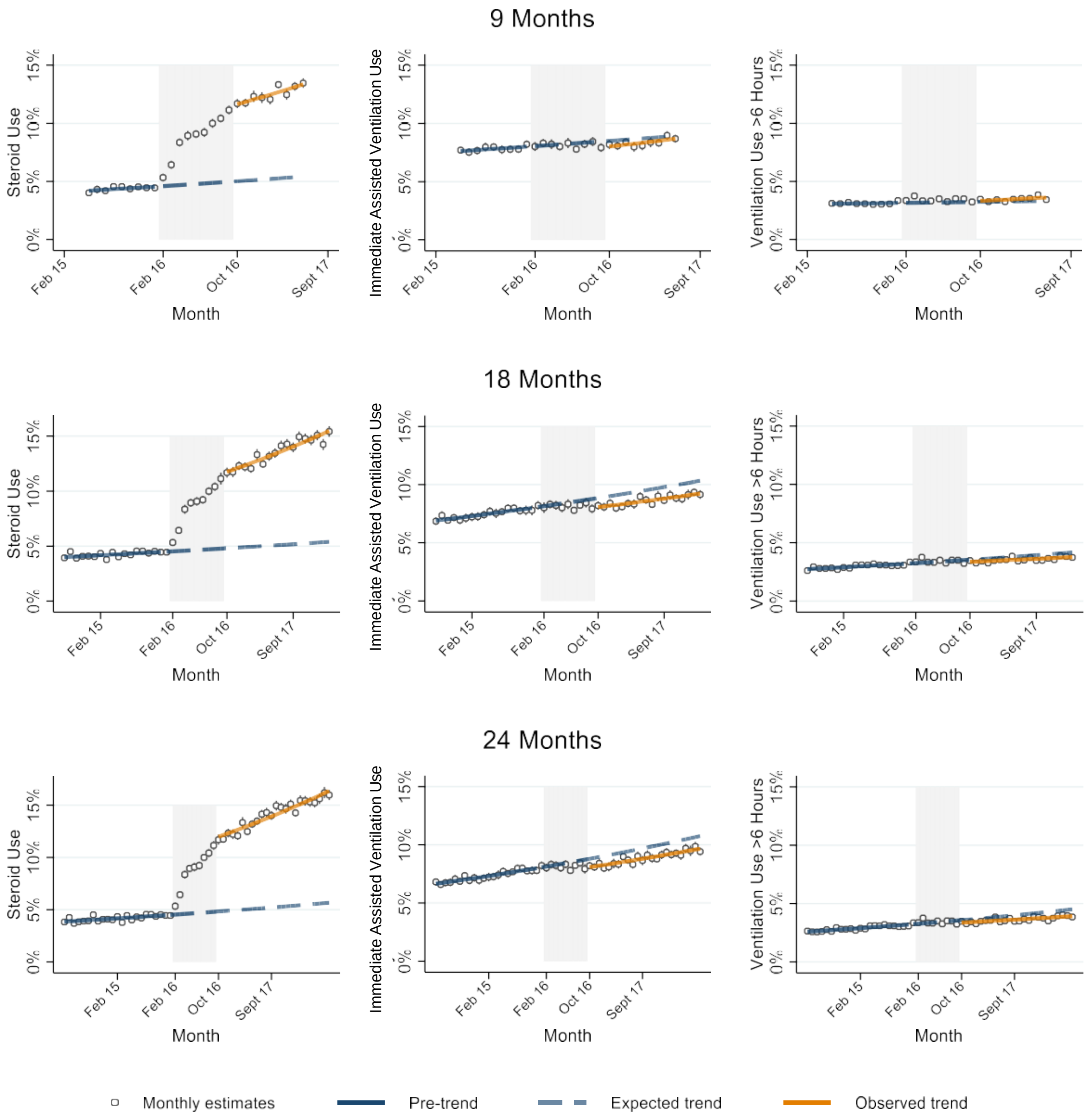

The adjusted models included the following covariates: completed weeks of gestation, maternal age, maternal race, maternal ethnicity, primary payer for birth encounter, primary delivering practitioner. The gray shaded area designates the trial dissemination period (February 2016-October 2016).

© 2022 Clapp MA et al. *JAMA Network Open.*

eTable 1. Adjusted incidence rate ratio estimates for the placebo tests in comparison to the effect estimated in the main analysis

Placebo Test Number	Steroid Use Adjusted IRR	Placebo Test Number	Assisted Ventilation Use Adjusted IRR
10	0.88	1	1.13
6	0.88	2	1.10
17	0.89	3	1.04
15	0.89	11	1.03
16	0.89	12	1.03
7	0.89	7	1.00
14	0.91	13	1.00
5	0.91	21	0.99
19	0.93	20	0.98
8	0.93	10	0.98
13	0.93	4	0.98
18	0.94	23	0.97
20	0.95	8	0.97
9	0.95	19	0.97
11	0.97	14	0.97
12	0.97	9	0.96
21	0.98	18	0.96
4	0.99	16	0.96
3	0.99	15	0.95
1	0.99	17	0.95
24	1.01	22	0.95
22	1.01	6	0.95
23	1.02	5	0.94
2	1.06	24	0.94
Main	**2.34**	Main	**0.91**
p-value	*0.04*	*p-value*	*0.04*

Placebo estimates are ranked from smallest to largest for steroid use and from largest to smallest for assisted ventilation use to be concordant with the direction of the observed association. The adjusted models included the following covariates: completed weeks of gestation, maternal age, maternal race, maternal ethnicity, primary payer for birth encounter, primary delivering practitioner.
IRR, incidence rate ratios.

© 2022 Clapp MA et al. *JAMA Network Open.*

eTable 2. Unadjusted and adjusted incidence rate ratios for steroid use with varying pre- and post-dissemination follow-up periods

Model	Unadjusted IRR	Adjusted IRR
Primary Analysis		
12 months	2.35 (2.18, 2.55)	2.34 (2.13, 2.57)
Sensitivity Analyses		
9 months	2.35 (2.12, 2.60)	2.32 (2.07, 2.60)
18 months	2.43 (2.29, 2.59)	2.45 (2.32, 2.59)
24 months	2.49 (2.37, 2.62)	2.48 (2.38, 2.60)

The adjusted models included the following covariates: completed weeks of gestation, maternal age, maternal race, maternal ethnicity, primary payer for birth encounter, primary delivering practicioner. IRR, incidence rate ratios.

eTable 3: Unadjusted and adjusted incidence rate ratios for immediate assisted ventilation use with varying pre- and post-dissemination follow-up periods

Model	Unadjusted IRR	Adjusted IRR
Primary Analysis		
12 months	0.93 (0.86, 1.00)	0.91 (0.85, 0.98)
Sensitivity Analyses		
9 months	0.96 (0.88, 1.06)	0.94 (0.87, 1.03)
18 months	0.91 (0.87, 0.95)	0.91 (0.87, 0.96)
24 months	0.92 (0.88, 0.96)	0.92 (0.88, 0.96)

The adjusted models included the following covariates: completed weeks of gestation, maternal age, maternal race, maternal ethnicity, primary payer for birth encounter, primary delivering practicioner. IRR, incidence rate ratios.

eTable 4: Unadjusted and adjusted incidence rate ratios for assisted ventilation use >6 hours use with varying pre- and post-dissemination follow-up periods

Model	Unadjusted IRR	Adjusted IRR
Primary Analysis		
12 months	0.99 (0.88, 1.12)	0.97 (0.84, 1.12)
Sensitivity Analyses		
9 months	1.06 (0.91, 1.23)	1.03 (0.88, 1.20)
18 months	0.94 (0.88, 1.01)	0.95 (0.87, 1.04)
24 months	0.94 (0.88, 1.00)	0.94 (0.88, 1.01)

The adjusted models included the following covariates: completed weeks of gestation, maternal age, maternal race, maternal ethnicity, primary payer for birth encounter, primary delivering practicioner. IRR, incidence rate ratios.

© 2022 Clapp MA et al. *JAMA Network Open.*
